# Simulation-based clinical assessment identifies threshold competence to practise physiotherapy in Australia: a crossover trial

**DOI:** 10.1186/s41077-022-00215-2

**Published:** 2022-07-27

**Authors:** Penny Moss, Anton Barnett-Harris, Darren Lee, Kriti Gupta, Shane Pritchard, Natalie Sievers, Maxine Te, Felicity Blackstock

**Affiliations:** 1 Australian Physiotherapy Council, Richmond, Australia; 2 Physiotherapy, School of Health Sciences, Western Sydney University, Locked Bag 1797, Penrith, NSW 2751 Australia

**Keywords:** Simulation-based assessment, High-stakes assessment, Physiotherapy, Registration, License, International

## Abstract

**Background:**

Although evidence exists for the efficacy of high-fidelity simulation as an educational tool, there is limited evidence for its application in high-stakes professional threshold competency assessment. An alternative model of simulation-based assessment was developed by the Australian Physiotherapy Council (APC), using purpose-written standardised patients, mapped to the appropriate threshold level. The aim of this two-phase study was to investigate whether simulation-based clinical assessments resulted in equivalent outcomes to standard, real-life assessments for overseas-trained physiotherapists seeking registration to practice in Australia.

**Methods:**

A randomised crossover trial comparing simulation-based assessment to real-life assessment was completed. Participants were internationally trained physiotherapists applying for registration to practice in Australia, voluntarily recruited from the Australian Physiotherapy Council (APC) assessment waiting list: study 1 *n* = 25, study 2 *n* = 144.

Study 1 participants completed usual APC real-life assessments in 3 practice areas, completed on different days at APC partner healthcare facilities. Participants also underwent 3 practice area-matched simulation-based assessments, completed on the same day at purpose-designed simulation facilities. Study 2 participants completed 3 simulation-based assessments and 1 real-life assessment that was randomly allocated for order and practice area. Assessment of competency followed the standard APC procedure of 90-minute examinations using The Moderated Assessment Form (MAF).

**Results:**

The overall pass rate was higher for real-life assessments in both studies: study 1, 50% versus 42.7%; study 2, 55.6% versus 44.4%. Chi-square analysis showed a high to moderate level of exact matching of pass/fail grades across all assessments: study 1, 73.4% (*p* < 0.001); study 2, 58.3% (*p* = 0.027). Binary logistic regression showed that the best predictors of real-life pass/fail grade were simulation-based MAF pass/fail grade (study 1, OR 7.86 *p* < 0.001; study 2, OR 2.037, *p* = 0.038) and simulation-based total MAF score (study 1, OR 1.464 *p* < 0.001; study 2, OR 1.234, *p* = 0.001).

**Conclusion:**

Simulation-based assessment is a significant predictor of clinical performance and can be used to successfully identify high stakes threshold competence to practice physiotherapy in Australia.

## Background

Physiotherapists who have trained overseas must be registered with the Physiotherapy Board of Australia to practice in Australia. When seeking registration, all applicants must demonstrate competence to practice through the completion of a written examination and three clinical assessments. All assessment procedures are administered by the Australian Physiotherapy Council (APC), as entrusted by the Physiotherapy Board of Australia.

The APC receives approximately 450 new applications per annum. Until 2018, all clinical assessments were undertaken at healthcare sites that were usually tertiary hospitals. A candidate would then be allocated whichever patient was available on the scheduled assessment day. Subsequently, the provision of clinical assessments depended upon the goodwill of individual facilities, the availability of patients and the availability of assessors. This presented complex and resource intensive challenges for assessment administrators as they ensured that assessments were timely, appropriate and consistent between locations. Given the necessarily pragmatic nature of this process, there was no guarantee that selected patients would present with conditions that were appropriate for entry-level physiotherapy competency assessment nor that across the three assessments all competencies could be assessed. This resulted in lengthy waiting times for candidates to be allocated, in addition to loss of earnings and potential skill regression.

In nursing and allied health professions, simulation-based education has been used implemented at undergraduate and postgraduate levels, with demonstrated benefits for both students and patients [1–6]. Challenges similar to those experienced by the APC have been observed in the clinical component of these degrees, with clinician goodwill required to support clinical learning in an increasingly complex and burdened healthcare environment. Entry-level programmes overcame these issues using simulation-based clinical education and work placements. Several large studies in nursing, physiotherapy and occupational therapy have shown that a high-fidelity simulation-based clinical placement, where actors portray patients in a purpose-designed realistic setting, can be used to replace standard ’real-life’ placements with no loss of competency [7–9].

Although simulation is now widely used for educational purposes across all medical professions, there is some disparity in its application for high-stakes assessments. In medicine, simulation has been used to assess high stakes competency for many years with over 45 papers identified in a scoping review published in 2021 [10]. For example, Isaac et al. [11] reported the validity and reliability of mixed-fidelity simulation (actors, part-trainers, mannequins, videos) for the assessment of milestones against national standards for anaesthesiologists by the United States Accreditation Council for Graduate Medical Education. More recently the effectiveness of simulation-based professional competency assessments has been reported for nursing [12, 13] and paramedicine [14]. However, the use of simulation for high stakes clinical assessment is rarely found in allied health. Gough et al. [15] surveyed all UK hospitals with intensive care units and found that, whilst 92% of cardiorespiratory physiotherapists had experienced simulation for educational purposes, only 39% had been assessed for competency using simulation. Simulation based assessment in Australian physiotherapy entry-level physiotherapy curricula, appears to be more widely used with 78% of Universities reporting using this methodology in assessments [16]. However, details on their use in high-stakes assessment was not provided in the study.

Despite strong evidence for the efficacy of simulation as an educational approach, there is limited evidence for its application in high-stakes assessment. Therefore, the aim of this two-phase study was to investigate whether simulation-based clinical assessments (referred to as simulation-based assessments) resulted in equivalent outcomes compared with standard, real-life assessments (referred to as real-life assessments) for overseas-trained physiotherapists seeking registration in Australia.

## Material and method

### Design

This study was a two-phase randomised crossover trial conducted at two sites: the APC Simulation Suite in Melbourne, Victoria (VIC), and the Western Sydney University (WSU) Simulation Clinic, Campbelltown, New South Wales (NSW). A crossover trial allowed each individual subject to act as their own control, as the variability in training and clinical experience prior to completing the assessment was deemed not measurable in a valid and reliable manner to allow for accurate matched controls. Further, as the results obtained in the high stakes examination were used to determine eligibility for registration as a practicing physiotherapist in Australia, it was not considered ethical to allocate individuals to an experimental assessment process to conduct a randomised controlled non-inferiority trial.

The first study phase (study 1), was a pilot study using a randomised crossover design, undertaken to explore ‘proof of concept’ and validate progression to a larger study. All participants underwent six clinical assessments: the three standard real-life assessments in the areas of cardiorespiratory, neurological and musculoskeletal practice plus three simulation-based assessments in the same three practice areas. Participants were randomly allocated to complete all real-life assessments or all simulation-based assessments first. The order of assessments was also randomised within each setting. Before consenting to participate, volunteers were informed that, as per normal APC procedures, only success in the three real-life assessments would be relevant for registration purposes. In order to reduce drop-out, participants only received their results once they had completed all six assessments.

**Fig. 1:**
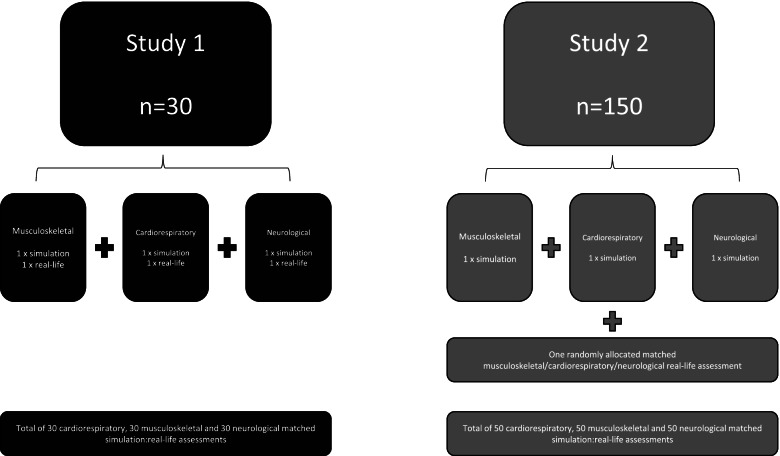
Overview of study design of the two phases of study

Following analysis of the pilot study, a larger follow-up study was undertaken in phase 2. In the second study, phase (study 2) volunteer participants completed three simulation-based assessments in randomised order, as in study 1, plus one real-life assessment, where the practice area was randomly allocated. The order of simulation or real-life assessment was also randomised as before. Success in the either the real-life assessments or simulation-based assessments would be relevant for registration purposes in the second study phase. Again, in order to reduce drop-out, participants only received their results once they had completed all assessments. Figure 1 illustrates the two phases of the research.

### Participants

For each phase of the study, candidates on the APC clinical assessment waitlist were invited to participate voluntarily at either the VIC or NSW locations. Invitations were offered in wait-list order to batches of 50 candidates until the sample size for each study phase was reached (study 1 = 30 participants, study 2 = 150 participants). The sample was a sample of convenience. Candidates who had already completed at least one real-life assessment or who required a second attempt at an assessment were excluded from the study. In order to manage any perceived power issues, email invitations, information sheets and consent forms were sent from the lead investigators who were not involved in APC administrative procedures. Once a volunteer returned their signed consent form, their name was passed on to APC staff for scheduling of real-life and simulation-based assessments.

### Study 1

#### Intervention

*Real-life assessments* followed the well-established standard APC procedure [17]. Designated APC staff liaised with partner healthcare facilities in VIC and NSW to schedule three assessments for each candidate. Each assessment was on a different day and likely to be at three different facilities. Clinical staff organised a patient for the candidate to assess and treat. Following standard APC procedure, the candidate’s performance was assessed by two clinician assessors, experienced in the practice area and in APC assessment procedures. Each real-life assessment followed standard APC timing: 10 min reading time; 5 min verbal summary; 50 min assessment and treatment; 10 min oral clarification. To ensure duty of care to the patient, APC procedure includes the option for an assessor to pause timing if they consider there is a potential safety problem. The candidate may be prompted, and the assessment continued or terminated, depending on the degree of safety concern.

*Simulation-based assessments* followed the same procedure as standard hospital-based assessments. Candidates were assessed using the same outcome measures by two expert clinicians who were experienced APC assessors and participating voluntarily. Both assessors were present throughout every assessment, applying assessment criteria simultaneously but independent. Assessors underwent additional training in simulation-based assessment which re-enforced the application of assessment criteria and included practice of applying the assessment tool with discussion on ratings for criteria as a component to improve reliability across the group of assessors. Assessors nominated their area of assessment (cardiorespiratory, neurological and/or musculoskeletal) and were trained in application of criteria for their nominated area. Assessors only completed assessments for their nominated area of practice. APC staff ensured that no candidate was assessed by the same assessor twice to minimise confirmation and sub-conscious biases. Each simulation-based assessment followed standard APC timing as described above, although there was no pausing for safety prompts since patient duty of care was not applicable.

Although processes were the same, simulation-based assessment offered several differences to real-life assessment. Real-life assessment involved travel to multiple locations and so might be scheduled weeks apart. In contrast, the three simulation-based assessments could be completed by each participant on the same half day at either the VIC or NSW locations. Simulated patient cases were written specifically for the study, mapped to the Physiotherapy Board of Australia, Physiotherapy Practice Thresholds [18] and using a national peer-review process [19]. In order to mirror real-life and to reduce collusion, each simulation day involved a new set of three patient cases, with morning candidates being held in ‘quarantine’ until afternoon candidates had arrived. For each candidate, the three simulated patient cases were balanced for level of acuity, gender, age, cultural heritage and communication difficulty. Age-appropriate role-play actors, experienced in health-care simulation, were trained to perform the role of each patient by an experienced simulation trainer in either VIC or NSW. This 3-h training occurred in the week leading up to each simulation day, with the training based on best practice for preparing actors for simulated patient roles [20].

#### Outcome measures

Standard APC clinical assessment tools and procedures were used for all hospital-based and simulation-based assessments. The Independent Assessment Form (IAF) and Moderated Assessment Form (MAF) are APC-specific tools which were developed by expert clinician members of the APC Assessment Sub-committee and updated in 2015. Both tools comprise the same seven domains (communication, assessment, interpretation, plan creation, plan development and implementation, effectiveness and safety) which match the Physiotherapy Practice Thresholds statements. The Physiotherapy Thresholds are a series of statements that the Physiotherapy Board of Australia outline as the minimum competencies for practice as a registered/licensed physiotherapist in Australia or New Zealand. The APC Assessment Manual details criteria and performance indicators [21]. Each assessor completes their own IAF during the assessment session without discussion with the other examiner. Once the assessment has concluded, assessors then discuss their decisions for each domain and overall pass/fail, reaching a consensus decision, which is recorded on the final MAF. The APC procedures require that each MAF domain must be passed to achieve an overall pass.

For the purposes of data analysis, the pass/fail designations for the MAF were coded as 2 and 1 respectively. This allowed a total MAF score to be calculated (maximum 14, minimum 7) (Table 1). For the purposes of this study, the IAF designations for each domain were expanded from pass/fail to four options, in which assessors were trained: non-competent, borderline, competent and excellent. These were scored 1, 2, 3 and 4 respectively, giving a maximum IAF score of 28 and a minimum of 7 (Table 1). This change had no impact on primary MAF candidate outcome.

**Table 1:** Independent assessment form and moderated assessment form grade options and scores used for data analysis.

Outcome measure	Domain		Overall	
	Grade options	Score	Grade options	Score
Independent assessment form	Non-competent, borderline, competent excellent	1 2 3 4	Non-competent, borderline, competent excellent	1 2 3 4
Moderated assessment form	Pass	2	Pass	2
	Fail	1	Fail	1

### Study 2

#### Intervention

The same procedures for all assessments were used in study 2. Real-life and simulation-based assessments were scheduled and organised in the same manner. New simulated patient scenarios were developed for each simulation day, using the same processes of mapping, blue-printing, review and actor training.

#### Outcome measures

Expert APC assessors were recruited and trained in the same manner, with the same timing, outcome measures and procedures used as for study 1.

### Data analysis

For both studies, assessment data for matched core practice areas were analysed, using Statistical Package for Social Sciences (SPSS) (v21, IBM), with alpha set at *p* < 0.05. From study 1, since each candidate completed both simulation-based and real-life assessments in each area, meaning that data for all three core areas was available for each candidate. For study 2, each candidate contributed matched simulation-based and real-life assessment data for one core area only. Data from both studies were not normally distributed hence non-parametric analyses were applied.

The same analyses were completed for each study to evaluate equivalence between real-life and simulation-based assessments, with the MAF used as the primary outcome. Percentages of pass/fail and total MAF scores were compared for each core area and in combination. Chi-square analyses evaluated the exact matching of pass/fail outcomes between real-life and simulation-based assessments. Spearman’s correlation coefficients were calculated for total scores and binary logistic regression was applied to evaluate significant predictors of real-life assessment pass grades. Effect sizes with 95% confidence intervals (CI) were calculated.

Assessors’ IAF grade categories (1−4) were also compared for each study using Wilcoxon matched pair tests, in order to evaluate whether the degree of agreement between assessors before moderation was similar between real-life or simulation-based assessments.

## Results

### Study 1

Twenty-five participants (10 male, 15 female) completed study 1. Fifteen were in NSW and 10 were in VIC. A total of 75 matched assessment datasets (25 per core area) were collected.

#### Pass/fail rates and total scores

The overall pass rates and total MAF scores for real-life and simulation-based assessments were similar (pass rates:50% and 42.7% respectively; MAF scores: real-life mean 11.91 (SD 2.73), simulation-based mean 11.19 (SD 2.43), *p* = 0.036), effect size Cohen’s *d* = − .268 (95% CI − .516 to − 0.018).

Participants were dichotomised post-hoc into those who passed or failed their real-life assessments. Those who passed their real-life assessments scored significantly higher in their overall simulation-based MAF assessment (pass 12.44 (SD 2.33), fail 9.94 (SD 2.55); *t* = − 4.095, *p* < 0.001, effect size (Cohen’s *d*) = − 1.11, 95% CI − 1.64 to − .578)). When practice areas were analysed separately, musculoskeletal assessments showed the greatest difference in simulation-based MAF scores between real-life pass/fail groups (pass 12.58 (SD 2.09), fail 8.89 (SD 2.58); *t* = − 3.517, *p* = 0.002, effect size (Cohen’s *d*) = − 1.35 (95% CI − 2.31 to − .386)).

#### Correlations between MAF scores

Spearman’s correlation coefficient (two-tailed) showed a moderate to good correlation between total MAF scores for real-life and simulation-based assessments across all assessments (*r* = 0.506, *p* < 0.001), as well as individually: cardiorespiratory (*r* = 0.469, *p* = 0.028); neurology (*r* = 0.508, *p* = 0.019); musculoskeletal (*r* = 0.597, *p* = 0.004).

#### Binary logistic regression predictors of hospital-based MAF scores

Binary logistic regression analysis found that simulation-based MAF pass/fail outcome was the best predictor of whether a candidate passed or failed their real-life assessment, with an odds ratio of 7.857 and moderate effect size (Table 2). Simulation-based MAF score and location of assessment were also significant predictors but with lower odds ratios of 1.464 and 0.358 respectively and very small effect sizes. Practice area was not a significant predictor (*p* = 0.648).

**Table 2:** Study 1: logistic regression analysis of simulation as a predictor of performance in real-life assessment of competency

	***B***	S.E.	Wald	Sig.	Exp(***B***)	95% CI Exp(***B***)	Effect size (Cohen’s ***f***^**2**^)
Sim-based MAF P/F^a^	2.061	0.573	12.943	0.000	7.857	2.556–24.154	0.568
Sim-based MAF total	0.381	0.109	12.15	0.000	1.464	1.181–1.813	0.105
Location	− 1.026	0.518	3.928	0.048	0.358	0.130–0.989	− 0.283
Area of practice	− 0.140	0.306	0.209	0.648	0.870	0.478–1.583	− 0.038

^a^Moderated assessment form pass/fail

#### Equivalence in assessor IAF grades

There was no significant difference in the grades given to a candidate independently by each of the two assessors for either the real-life (assessor 1 mean 16.55 (SD 6.20); assessor 2 mean 17.18 (SD 5.28), *p* = 0.214) or simulation-based assessments (assessor 1 mean 16.05 (SD 5.71); assessor 2 mean 15.43 (SD 5.66), *p* = 0.105). There was acceptable exact agreement for real-life assessments (82%) and for simulation-based assessments (78.4%).

### Study 2

Study 2 was completed by 144 participants, 67 in NSW and 77 in Victoria (Table 3). Forty-eight matched assessments were provided for each core practice area (see supplementary information).

**Table 3:** Study 2: participant demographic data

Gender (male:female)	44:100	
Age (mean, range)	32.8 (25–57) years	
Years since physio qualification (mean, range)	9.3 (0–26) years	
Geographic location of origin/qualification (%)	Indian subcontinent	51.4%
	Europe	13.9%
	Philippines	13.7%
	Middle East	10.4%
	Africa	4.5%
	Americas	3.5%
	Asia	2.6%

#### Pass/fail rate and total scores

As in study 1, the overall pass rate for real-life assessments was higher than for simulation-based assessments (55.6% and 44.4% respectively) although total MAF scores were similar (real-life mean MAF 12.02 (SD 2.53), simulation-based mean 11.47 (SD 2.67); *t* = − 2.098, *p* = 0.038; small effect size (Cohen’s *d* = – .174 (95% CI –.337 to –0.010).

Similarly, participants who passed their real-life assessment scored significantly higher in their simulation-based assessment (*t* = – 3.356, *p* = 0.001): real-life pass mean 12.11 (SD 2.41), real-life fail mean 10.66 (DS2.79); moderate effect size (Cohen’s *d*) = – .579 (95% CI – .911 to – 0.245. Musculoskeletal assessments again showed the greatest discrimination (real-life pass 11.93 (SD 2.66), real-life fail 9.57 (SD 2.36); *t* = – 3.196, *p* = 0.003); large effect size (Cohen’s *d*) = – .930 (95% CI – 1.526 to – 0.324).

#### Correlations between MAF scores

There was a small but significant correlation between total MAF scores for real-life and simulation-based assessments when combined for all areas (*r* = 0.279, *p* = 0.001), although only musculoskeletal assessments showed an individually significant correlation (*r* = 0.428, *p* = 0.002). Cardiorespiratory and neurology assessments did not show significant correlations (*r* = 0.226 (*p* = 0.122) and *r* = 0.128 (*p* = 0.385) respectively).

#### Binary logistic regression predictors of real-life MAF scores

Simulation-based pass/fail outcome, simulation-based assessment MAF total score and location of assessment were found to be significant predictors of real-life pass/fail, with odds ratios of 2.037, 1.234 and 0.818 respectively, although all effect sizes for each were very small. Practice area was not a significant predictor (*p* = 0.681) (Table 4).

**Table 4:** Study 2: logistic regression analysis of simulation as a predictor of performance in real-life assessment of competency

	***B***	S.E.	Wald	Sig.	Exp(***B***)	95% CI Exp(***B***)	Effect size (Cohen’s ***f***^**2**^)
Sim-based MAF P/F^a^	0.711	0.342	4.320	0.038	2.037	1.041–3.984	0.196
Sim-based MAF total	0.210	0.066	10.207	0.001	1.234	1.085–1.403	0.058
Location	– 0.201	0.337	0.357	0.550	0.818	0.422–1.583	– 0.055
Area of practice	– 0.084	0.206	0.169	0.681	0.919	0.614–1.375	– 0.023

^a^Moderated assessment form pass/fail

#### Equivalence in assessors’ IAF grades

As in study 1, there was good agreement between independent assessors for both real-life and simulation-based assessments: no significant difference in the grades given by each of the assessors for real-life (*p* = 0.217) or simulation-based (*p* = 0.748). There was 83.3% exact agreement for real-life assessments and 81.1% for simulation-based assessments.

## Discussion

This is the first study to investigate high-fidelity simulation-based assessment in high stakes physiotherapy clinical competency assessment. This two-part randomised crossover study revealed a good level of equivalence in pass/fail and total scores between real-life and simulation-based assessments: pass/fail rates and total scores were similar; there was a good level of exact matching of pass/fail grades (73.4% for Study 1 and 58.3% for study 2); and simulation-based assessment score was a significant predictor of real-life pass/fail outcome.

Whilst previous studies in medical, nursing and paramedical contexts have reported validating simulation for competency assessments, this is most often a mix of part-trainer, mannequin, video and paper-based simulation experiences completed in an objective structure clinical examination [4, 10]. To our knowledge, no previous healthcare profession accreditation body or university have validated a simulation-based assessment method which covers the entire spectrum of a patient interaction from assessment to intervention to discharge planning using actor portrayal of a patient case in a single time, thereby replicating the full clinical encounter*.*

The degree of equivalence between real-life and simulation-based assessments was good rather than excellent, which is an outcome that might raise concerns about the validity of simulation as an alternative in high stakes assessment. However, complete equivalence was unlikely to have been achieved, even within the same practice area. Although all procedures were standardised, the patients available to candidates in healthcare facilities could not be standardised, and so matching real-life and simulation cases was unrealistic. However, a post-hoc comparison of patient cases demonstrated unexpected but clear differences between the two assessment settings, with simulation providing greater variety in conditions and levels of acuity. This difference would inevitably limit equivalence in assessment scores.

As simulation intentionally controls the distractions and complexities of a healthcare environment, simulation-based assessment could be considered to be “easier” than real-life assessment. The unpredictable nature of real-life assessment should challenge participants to respond to unexpected complexities and potentially highlight when a person does not meet the threshold competencies more readily. In contrast to this theory, the current study found that the pass rate for real-life assessment was consistently higher than for simulation-based assessment. The lack of real-life distractions may in fact benefit the rigour of the assessment process rather than the candidate, allowing competent or non-competent performances to be more easily identified. The assessors themselves are not distracted by disruptions and are able to focus solely on the performance of the candidate. Further, the simulation-based assessment intentionally controls what occurs during the assessment process so sufficient complexities can be introduced. For example, the assessor in simulation does not have to be alert to unforeseen potential adverse events. Instead they can allow a session to unfold uninterrupted and can observe how a candidate manages any safety problem that has been scripted. An assessment does not need to cease due to safety of the “patient” and poor performance clearly demonstrated for confidence in the application of the assessment criteria.

Aside from confirming competency and “grading”, the simulation-based assessment process has demonstrated a range of added benefits. The system is considerably more efficient for staff, for assessors and for candidates. All assessments are completed in the same day, at the same location with only APC staff involved in administration. The whole process is more equitable, with minimisation of interstate variations in healthcare practice or interpretation of assessment criteria. Clinical cases are standardised and written at an appropriate level for assessment of threshold competency. Mapping ensures that all threshold competencies can be demonstrated across the three cases for each candidate, which is impossible in real-life assessments. There is opportunity for assessors to develop a more consistent approach to decision-making across practice areas, those within the same practice area assessing up to six candidates in a day, alongside colleagues from the other practice areas. Previously assessors were likely to only assess one or two candidates in a day and would not have had contact with colleagues from other practice areas. The increased cohesion between assessors is reflected in the high level of agreement (81%) in IAF grades for study 2.

There is one significant limitation to these studies that needs to be considered in interpreting the results. Since simulation-based assessment was compared with the standard real-life APC clinical assessment process, the existing APC assessment tools were used. Although, these tools are well-established, created from nationally agreed Physiotherapy Practice Threshold [15] [competency] statements and found to have content validity, they have not yet undergone specific processes to rigorously determine psychometric properties. Indeed evaluation of the validity and reliability of assessment tools is considered best practice in healthcare simulation by the International Nursing Association of Clinical and Simulation Learning [22]. In such research, the use of a structure framework for validating the assessment process as well as tool should be considered. Validation frameworks improve the rigour of a study by supporting the selection and collection of evidence and identification of shortcomings in the research methodology and assessment process being designed [23]. Many framework’s exist, however, Kane’s Framework [24, 25] has been appropriately applied and demonstrated to support the validation process in simulation-based assessments of clinical competency [26]. Kane’s Framework identifies four key inferences in generating useful interpretations: scoring, generalisation, extrapolation and implications/decisions. This study has examined generalisation (through mapping of the scenarios against the Physiotherapy Practice Thresholds [competencies] for the simulation-based assessment), extrapolation (through comparison of the simulation-based assessment with real-life assessment) and implications through examining the number of pass/fail scores and participants who successfully we able to register as practicing physiotherapists in Australia). The study has not examined the scoring component thoroughly. Steps to improve the application of scoring and thereby reliability and validity were taken, but all shortcomings were not addressed. Considering that this is a high-stakes assessment, use of a validity framework for evaluation is a critical next step for furthering our understanding of application of simulation-based assessment. Future research validating the APC assessment tools is required, covering all aspects of validation of an assessment process as outlined in Kane’s Framework.

The results from this study have been translated into practice with the APC moving to a system of simulation-based clinical assessment only from April 2019. This has had significant positive anecdotal outcomes with wait times for assessment being reduced from over a year to under two months, a decrease in burden for healthcare sites in hosting assessments which in turn allows for other education focussed activities to increase, and participants having a sense of control over when their clinical assessment are scheduled as dates for assessments have more flexibility to be negotiated.

## Conclusions

This is the first randomised crossover study to explore the validity of high-fidelity simulation-based assessment for evaluating threshold competency in internationally-trained physiotherapists applying for Australian registration. Despite clear differences in setting and patient choice, a good level of equivalence was found between real-life and simulation-based assessments. Validity and reliability of assessment tools are an important component of high-stakes assessments using simulation and require further research using structured frameworks such as that proposed by Kane [23, 24]. The findings suggest that simulation, using purpose-written scenarios portrayed by trained actors, can be used to successfully identify threshold competence.

## Data Availability

The datasets used and/or analysed during the current study are available from the corresponding author on reasonable request.
